# Comprehensive characterization of functional eRNAs in lung adenocarcinoma reveals novel regulators and a prognosis-related molecular subtype

**DOI:** 10.7150/thno.47039

**Published:** 2020-09-14

**Authors:** Na Qin, Zijian Ma, Cheng Wang, Erbao Zhang, Yuancheng Li, Mingtao Huang, Congcong Chen, Chang Zhang, Jingyi Fan, Yayun Gu, Xianfeng Xu, Liu Yang, Xiaoxia Wei, Rong Yin, Yue Jiang, Juncheng Dai, Guangfu Jin, Lin Xu, Zhibin Hu, Hongbing Shen, Hongxia Ma

**Affiliations:** 1 Department of Epidemiology, Center for Global Health, School of Public Health, Nanjing Medical University, Nanjing 211166, China.; 2 Jiangsu Key Lab of Cancer Biomarkers, Prevention and Treatment, Collaborative Innovation Center for Cancer Medicine, Nanjing Medical University, Nanjing 211166, China.; 3 Department of Bioinformatics, School of Biomedical Engineering and Informatics, Nanjing Medical University, Nanjing 211116, China.; 4 Department of Thoracic Surgery, Jiangsu Cancer Hospital, Jiangsu Institute of Cancer Research, Nanjing Medical University Affiliated Cancer Hospital, Nanjing 210029, China.

**Keywords:** Lung adenocarcinoma, enhancer RNA, immune deficiency, prognosis, copy number amplification

## Abstract

**Rationale:** As the transcriptional products of active enhancers, enhancer RNAs (eRNAs) are essential for the initiation of tumorigenesis. However, the landscape and functional characteristics of eRNAs in Chinese lung adenocarcinoma, and the clinical utility of eRNA-based molecular subtypes remain largely unknown.

**Methods:** A genome-wide profiling of eRNAs was performed in 80 Chinese lung adenocarcinoma patients with RNA-seq data. Functional eRNAs and associated genes were identified between paired adenocarcinoma and adjacent samples. Unsupervised clustering of functional eRNAs was conducted and the associations with molecular characteristics and clinical outcomes were accessed by integrating whole-genome sequencing data and clinical data. Additionally, 481 lung adenocarcinoma patients were used for the validation based on The Cancer Genome Atlas (TCGA) dataset.

**Results:** A total of 3297 eRNAs with sufficient expression were identified, which were globally upregulated in adenocarcinoma samples compared to matched-adjacent pairs (*P =* 7.61×10^-3^). Further analyses indicated that these upregulated eRNAs were correlated with copy number amplification (CNA) status (Cor = 0.22, *P =* 0.045), and eRNA-correlated genes were primarily involved in cell cycle and immune system-related pathways. Based on the co-expression analysis of eRNAs with protein-coding genes, we defined 188 functional eRNAs and their correlated genes were overrepresented in cancer driver genes (ER = 1.98, *P =* 5.95×10^-12^) and clinically-actionable genes (ER = 2.19, *P =* 3.44×10^-4^). The eRNA-based consensus clustering further identified a novel molecular subtype with immune deficiency and a high-level of genomic alterations, which was associated with poor clinical outcomes of lung adenocarcinoma patients (OS: HR = 1.91, *P =* 0.015; PFI: HR = 1.64,* P =* 0.034).

**Conclusions:** The genome-wide identification and characterization of eRNAs reveal novel regulators for the development of lung cancer, which provides a new biological dimension for the understanding of eRNAs during lung carcinogenesis and emphasize the clinical utility of eRNA-based molecular subtypes in the treatment of lung adenocarcinoma.

## Introduction

Lung cancer is the leading cause of cancer-related mobility and mortality worldwide 1. Adenocarcinoma is the predominant histological subtype of lung cancer, accounting for approximately 40% of lung cancer cases 2, 3. Despite recent advances in multi-modality therapy, the overall 5-year survival rate of lung adenocarcinoma remains about 15% 3, mainly because of the late-stage diagnosis and a lack of effective therapeutic targets.

The development of lung adenocarcinoma is a multistep, evolving process which involves the interaction between environmental exposures and a diversity of molecular alterations, including germline variations, somatic variations, transcriptional and epigenetic alterations 4-9. Recently, by integrating multi-dimensional omics (multi-omics) data, The Cancer Genome Atlas (TCGA) group profiled lung adenocarcinoma and identified several molecular subtypes with targetable candidates in oncogenic pathways 7. However, because of the intra-tumor heterogeneity, biological mechanisms underlying the development and progression of lung adenocarcinoma remain elusive. Moreover, the application of multi-omics strategy in clinical practices still faces some challenges, such as the sample collection, the high cost, and the target therapy selection 10. Therefore, using a one-dimensional feature sharing interconnectedness with other omics as an alternative is essential for elucidating molecular mechanisms underlying the pathogenesis of lung adenocarcinoma, which may also provide candidate therapeutic targets and improve clinical outcomes.

Enhancers are a class of distal DNA *cis*-regulatory elements that can be activated by chromosomal rearrangement, focal amplification and over-expression of transcriptional factors (TFs) 11, which may selectively regulate genes during the development and differential of cancer cells 12-17. In the past decade, the Encyclopedia of DNA Elements (ENCODE) 18, Functional Annotation of the Mammalian Genome (FANTOM) 19 and Roadmap Epigenomics 20 projects have detected tens of thousands of enhancers across different cell types and tissues. Recently, mounting evidence shows that enhancers can also act as transcriptional units to produce enhancer RNAs (eRNAs), which are hallmarks of active enhancers 15, 21, 22. The functional importance of eRNAs in oncogene deregulation and cancer initiation have been established in many cancer types 15. For example, the activation of *MYC*-eRNAs was reported to promote the development of a range of cancers 23-25. 17β-estradiol (E2)-associated eRNAs activate the expression of E2-dependent genes in breast cancers 26. *KLK3*-eRNAs control the expression of androgen receptor-related genes in prostate cancers 26. eRNA *AC026904.1* was considered as one of the key regulators of EMT in metastatic breast cancer 27. Although some lung adenocarcinoma related eRNAs have been described in TCGA samples 28, 29, their transcriptional landscape, molecular characteristics, and clinical utility among Chinese lung adenocarcinoma remain largely unexplored.

Thus, by integrating whole-genome sequencing (WGS) and RNA-seq of 80 lung adenocarcinoma patients from Nanjing Lung Cancer Cohort (NJLCC), we intended to present a genome-wide detection and characterization of eRNAs in lung adenocarcinoma. The molecular characterization and prognostic value of our eRNA-based molecular subtypes were further evaluated among 481 TCGA lung adenocarcinoma patients.

## Materials and Methods

### Study subjects

Surgically resected tumor specimens, adjacent normal tissues, and matched peripheral blood samples of 80 Chinese lung adenocarcinoma patients were collected from Jiangsu Cancer Hospital affiliated to Nanjing Medical University in China, and were subjected to WGS and RNA-seq. All patients had definite pathological diagnosis and had no treatment or neoadjuvant therapy history before surgery. Frozen tumor-adjacent pair specimens were stained with hematoxylin and eosin, and microscopically evaluated by two independent pathologists. Only adenocarcinoma tissues with malignant cell purities over 70% and adjacent normal tissues contained no tumor cells were selected for DNA and/or RNA extraction and subsequent sequencing. The study was approved by Nanjing Medical University, and written informed consent were obtained from all participants. Sequencing data of 481 lung adenocarcinoma patients (55 with matched normal adjacent samples) from TCGA were also included in this study, all of whom were subjected to RNA-seq and 477 tumor-blood pairs were subjected to whole-exome sequencing (WES). Detailed demographic information of participants is shown in **Table S1**.

### RNA extraction and RNA sequencing

Total RNA was extracted from frozen tumor-adjacent tissue pairs using the RNeasy Mini Kit (Qiagen, Hilden, Germany) according to the manufacturer's instructions. The quality and quantity of extracted RNA were assessed using the NanoDrop 2000 (Thermo Fisher Scientific, Wilmington, DE, USA), Qubit 2.0 Fluorometer (Life Technologies, CA, USA), and 1% agarose gel electrophoresis. RNA integrity was accessed using RNA Nano 6000 Assay Kit (Agilent Technologies, CA, USA) and only high-quality RNAs (RIN ≥ 7.5) were selected for cDNA library construction.

A total amount of 3 μg high-quality RNA per sample was used for ribosomal RNA removal by Epicentre Ribo-zero rRNA Removal Kit (Epicentre, USA). Sequencing libraries preparation with the rRNA depleted RNA was performed with NEBNext Ultra Directional RNA Library Prep Kit for Illumina (NEB, USA) following manufacturer's recommendations. Clustering of the index-coded samples was performed on a cBot Cluster Generation System using TruSeq PE Cluster Kit v3-cBot-HS (Illumina, San Diego, CA, USA) followed by 150-bp paired-end sequencing on the HiSeq 1500 platform (Illumina, San Diego, CA, USA) according to the manufacturer's protocols. The sequenced reads of 80 lung adenocarcinoma tumor-adjacent pairs are shown in **Table S2**.

### Annotation and quantification of eRNAs

We first obtained the genomic regions of 1,310,152 candidate regulatory elements defined by Chip-seq histone modification peaks from the ENCODE consortium (https://www.encodeproject.org/), and 65,423 active enhancers defined by the integration of chromatin modification, transcription factor binding, and CAGE-seq data from FANTOM5 19, and then filtered out enhancers that can only be detected in one dataset. Finally, a total of 48,453 enhancers were included, including 1932 exonic enhancers (enhancer regions overlapped with the exon regions of known genes), 18,501 intergenic enhancers (enhancers located in the intergenic regions), and 28,020 intronic enhancers (enhancers regions overlapped with the intronic regions of known genes) (**Table S3**).

For enhancer expression quantification, RNA reads were first aligned to the GENCODE v19 genome assembly with STAR v2.4.1 30, and then quantified with featureCounts v1.5.0 31. Only enhancers with raw read counts > 1 in more than 10% tumor or adjacent normal samples were defined as transcribed eRNAs and were included in the following analysis. Expression of eRNAs was normalized to the number of reads per million mapped reads (RPM) 28. The quality score and base-call distributions of raw sequencing reads were accessed with the FastQC tool (http://www.bioinformatics.babraham.ac.uk/projects/fastqc).

### Whole-genome sequencing and variants detection

Paired-end WGS (150 bp) was performed on 80 matched tumor-blood lung adenocarcinoma samples. Detailed methods for DNA extraction and WGS have been described in our previous study 32. The quality score and base-call distributions of raw sequencing reads were accessed with the FastQC tool (http://www.bioinformatics.babraham.ac.uk/projects/fastqc). The Burrows-Wheeler Aligner (BWA-MEM) algorithm (http://bio-bwa.sourceforge.net/) was used to map sequenced reads to the reference genome (GRCh37) with default parameters 33, and Picard (v1.70, http://broadinstitute.github.io/picard/) was used to mark the duplicates which were discarded from further analyses. Local realignment and base quality score recalibration (BQSR) were performed with the Genome Analysis Toolkit (GATK, version 3.7) with default settings 34.

Somatic single-nucleotide variations and small insertions and deletions were detected using the Mutect2 mode in GATK following the best practice (https://software.broadinstitute.org/gatk/best-practices/). Somatic variants were further filtered out if it was detected in: (1) a panel of normal built by the 80 matched normal samples; (2) the segmental duplication or simple repeat regions marked by UCSC browser (http://genome.ucsc.edu/); or (3) the 1000 genomes project (the Phase III integrated variant set release, across 2,504 samples) with the same mutation direction.

### DNA-seq and RNA-seq data from TCGA project

Raw Illumina HiSeq RNA-seq data of 481 unduplicated lung adenocarcinoma samples was downloaded from the GDC data portal (https://portal.gdc.cancer.gov/) in TCGA and was performed with the same quantification process as NJLCC data. The mRNA expression data of 481 adenocarcinoma samples was obtained from the UCSC Xena website (https://xenabrowser.net/datapages/) and quantified as fragments per kilobase of exon per million reads mapped (FPKM). To replicate the associations of eRNA-based molecular subtypes with somatic mutations and copy number alterations identified in our data, we further obtained the somatic mutation data of TCGA lung adenocarcinoma samples from a recent published study 35, and the somatic copy number alteration information from the cBioPortal website 36, 37.

### Comparison of eRNA expression between tumor and adjacent normal samples

The difference of each eRNA expression (single-eRNA) and global eRNA expression (global-eRNA) between 80 adenocarcinomas and matched adjacent normal samples was evaluated. For global comparison, we first measured the global eRNA expression by counting RPM on all expressed eRNAs for each sample, and then scaled the expression by the number of expressed eRNAs. Wilcoxon signed-rank test was used to perform the differential expression analysis. For single-eRNA level analysis, log_2_ transformed fold change was further calculated to quantify the expression change from tumor to normal samples.

### Integrative analysis of eRNA expression with other demographic and molecular characteristics

For demographic characteristics, Wilcoxon rank-sum test was performed to evaluate the difference of global-eRNA expression in subgroups divided by age, gender, or smoking status. For genomic variation evaluation, we first estimated the percentage of genome that was affected by copy number gains (the fraction of amplified genome) or losses (the fraction of deleted genome) 36, and the number of non-silent mutations for each sample, and then performed Spearman's rank correlation test to evaluate the correlation between global-eRNA expression and two molecular characteristics (copy number variation and non-silent mutations). Fisher's exact test was used to evaluate the association between mutation status of previously reported lung adenocarcinoma significantly mutated genes and global-eRNA expression level. The same analysis was performed to estimate the correlation between the copy number alteration status of previously reported copy number variation genes of lung adenocarcinoma and global-eRNA expression level.

### Co-expression analysis and Gene Set Enrichment Analysis (GSEA)

To evaluate the difference of biological functions of transcribed eRNAs in four groups of samples (tumor and normal tissues of smokers or non-smokers), we first conducted co-expression analysis of eRNAs and 20,345 protein-coding genes (PCGs) defined in the GENCODE dataset (https://www.gencodegenes.org/, Version 19), and computed Spearman's rank correlation coefficient for all eRNA-PCG pairs. Then, we performed GSEA in above four groups of samples based on the GO Biological Process Ontology gene sets, KEGG, and Reactome pathway databases with the R Bioconductor package clusterProfiler (v 3.10.1) 38, respectively. All PCGs were ranked according to the number and the average correlation coefficient of co-expressed eRNAs. eRNA-PCG pairs with absolute correlation coefficient ≥ 0.2 and the Benjamini-Hochberg false discovery rate (FDR-BH) adjusted *P*-value < 0.05 were defined as co-expressed.

### Definition of functional upregulated/downregulated eRNAs in lung adenocarcinomas

**Figure 1:**
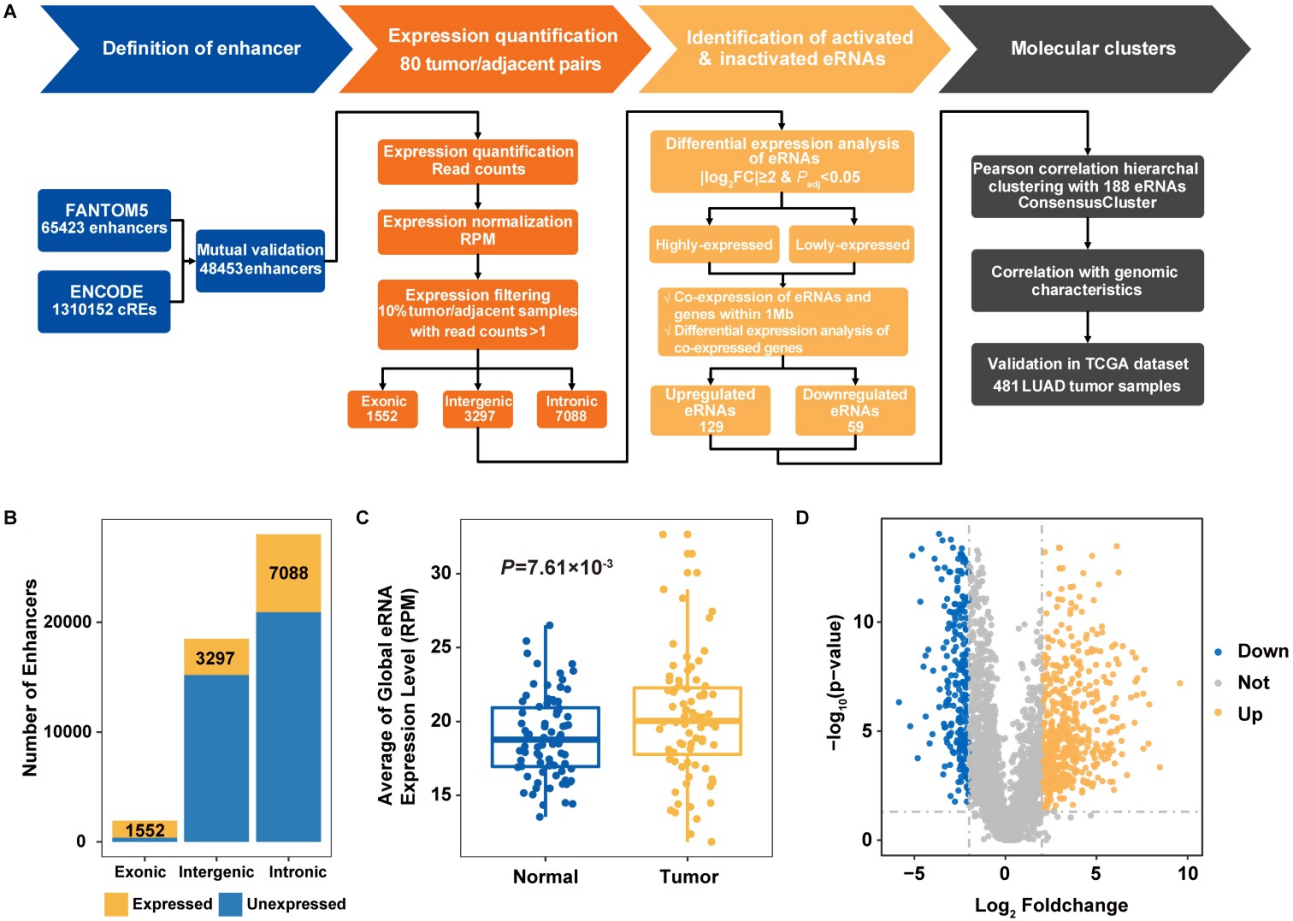
** Identification of transcribed eRNAs in Nanjing Lung Cancer Cohort (NJLCC) lung adenocarcinomas. A.** Overview of the study workflow. **B.** Number of transcribed eRNAs in 80 tumor/adjacent lung adenocarcinoma (LUAD) samples. **C.** Global expression of transcribed eRNAs in tumor and adjacent samples. **D.** Differential expression pattern of transcribed eRNAs in tumor and adjacent samples.

To define candidate functional eRNA-PCG pairs in lung adenocarcinoma, we evaluated the expression alterations of both eRNAs and co-expressed PCGs in tumor and adjacent normal samples. Putative eRNA-PCG pairs were defined if matching all the following criteria: (1) eRNA with a significantly elevated (upregulated eRNA: log_2_FC ≥ 2, *P_FDR_ <* 0.05) or decreased (downregulated eRNA: log_2_FC ≤ -2, *P_FDR_ <* 0.05) expression in tumor samples; (2) the co-expressed PCG showed a positive expression correlation with specific eRNA in tumor (upregulated eRNA) or normal (down-regulated eRNA) samples within one mega base pair (Mbp) (the length scale was restricted to avoid spurious predictions); and (3) the co-expressed PCG with a significantly upregulated (Mean_tumor_ ≥ 0.5 Transcripts Per Kilobase of exon model per Million mapped reads [TPM], log_2_FC ≥ 2, *P_FDR_ <* 0.05) or downregulated (Mean_normal_ ≥ 0.5 TPM, log_2_FC ≤ -2, *P_FDR_ <* 0.05) expression pattern in tumor samples (**Figure 1A**).

To further identify functional eRNA-PCG pairs activated by the copy number amplification (CNA) of target eRNA regions, an eRNA was included if met all the following three criteria: (1) eRNA with an amplification ratio ≥ 10%; (2) the correlation coefficient between the copy number and expression level of the candidate eRNA ≥ 0.2 and the correlation* P <* 0.05; and (3) association between the candidate eRNA and co-expressed PCG was independent of the copy number level of the co-expressed PCG.

### Consensus clustering of eRNA expression profile

To further distinguish subgroups of samples sharing similar expression patterns of eRNAs, consensus clustering was applied with the R package ConsensusClusterPlus (v 1.46.0) 39. The input data for each sample was the expression value (RPM) for above-defined functional eRNAs. Expression level of each eRNA was mean-centered across the samples prior to clustering. The following parameters were used for consensus clustering: number of repetitions = 1000; pItem = 0.7; pFeature = 0.7; Pearson distance metric and Ward linkage method.

To infer biological functions of the differentially expressed PCGs in above defined subgroups of samples, we conducted GSEA analysis using log_2_ transformed fold change of PCGs based on the GO Biological Process Ontology gene sets, KEGG, and Reactome pathway databases with the R Bioconductor package clusterProfiler (v 3.10.1) 38, respectively.

### Survival analysis

To evaluate the prognosis effect of eRNA-based clusters, follow-up data were obtained for TCGA lung adenocarcinoma patients 40. The multivariate Cox proportional hazards regression model was performed with adjustment for age, gender, and smoking status, where crude hazard ratios (HRs) and 95% confidence intervals (CIs) were calculated. Participants with a follow-up time less than one month were not included. Overall survival (OS), disease-free interval (DFI), and progression-free interval (PFI) were set as clinical outcome endpoints, respectively. The Kaplan-Meier (K-M) method was used to create survival plots and log-rank test was used to compare the difference of survival curves.

### GRO-seq data

The GRO-seq data of a lung adenocarcinoma cell line A549 41 was retrieved from Gene Expression Omnibus (GEO) (GEO Accession: GSE92375). Details for cell line culture and libraries preparation as well as the pipeline of processing and mapping of the sequencing data were described in a previous work^41^ . The* de novo* identification of enhancers was performed using R package groHMM (v1.16.0) with default parameters 42.

### RNA extraction and qRT-PCR (Quantitative Real-time PCR) analyses for eRNA

Total RNA was extracted from lung adenocarcinoma tissues using Trizol reagent (Invitrogen). The expression of candidate eRNAs were determined by using qRT-PCR. RNA was reverse transcribed to cDNA by using a Reverse Transcription Kit (Takara, Dalian, China), and qRT-PCR analyses were performed with SYBR Green (Takara, Dalian China). The results were normalized to the expression of GAPDH. The qRT-PCR and data collection were carried out on ABI 7500 real-time PCR system (Applied Biosystems, USA). The specific primer sequence for eRNA was designed according to the reference sequence of genome (hg19). The sequence of primers was listed in **Table S4**.

### Statistical analysis

Expression correlations between eRNAs and *EP300* and *POLR2A* were evaluated by Spearman's rank correlation test. Two-sided *P* values < 0.05 were considered statistically significant. General statistical analyses were performed using R software (R version 3.3.1, The R Foundation for Statistical Computing, http://www.cran.r-project.org/).

## Results

### Overview of eRNA expression in lung adenocarcinoma

We comprehensively profiled the expression signal of 48,453 previously annotated enhancers (FANTOM5 and ENCODE) with RNA-seq data from 80 NJLCC lung adenocarcinoma tumor-adjacent normal pairs (**Figure 1A, Figure S1**), and detected a total of 11,937 eRNAs with expression across more than 10% of the samples, including 1552 exonic, 3297 intergenic, and 7088 intronic eRNAs (**Figure 1B, Table S3**). We included 3297 intergenic eRNAs with a median length of 699 bp (75~6598 bp) in the following analysis to avoid the influence of transcribed genes.

To evaluate the transcriptional activity of transcribed eRNAs, we compared the chromatin status of 3297 transcribed and 15,204 un-transcribed intergenic enhancers with 44 TF binding site annotations of two well-known TFs associated with transcriptional activity (POLR2A and EP300) from 11 types of cell lines, and observed an enrichment of transcribed eRNAs in the binding sites of these two TFs (**Table S5**). Further expression correlation analysis also revealed that the number of positively-correlated eRNAs for *EP300* and *POLR2A* (*EP300*: 430/3297=13.04%, ER = 4.00, *P =* 9.17×10^-46^; *POLR2A*: 511/3297=15.50%, ER = 4.33, *P =* 6.32×10^-58^) was significantly higher than that of negatively-correlated eRNAs (*EP300*: 119/3297=3.61%; *POLR2A*: 134/3297=4.06%) (**Table S6, Figure S2**). In addition, we also found that tumor samples had significantly elevated eRNA expression at both the global-level (expression of all eRNAs as a combination) (*P =* 7.61×10^-3^) (**Figure 1C**) and individual-level (expression of every single eRNA) than adjacent normal samples, where 15.68% (517/3297) of the eRNAs with higher expression in tumor samples and 7.19% (237/3297) in adjacent samples (**Figure 1D**).

### Tobacco smoking exposure affects the epigenetic regulation of eRNA expression

**Figure 2:**
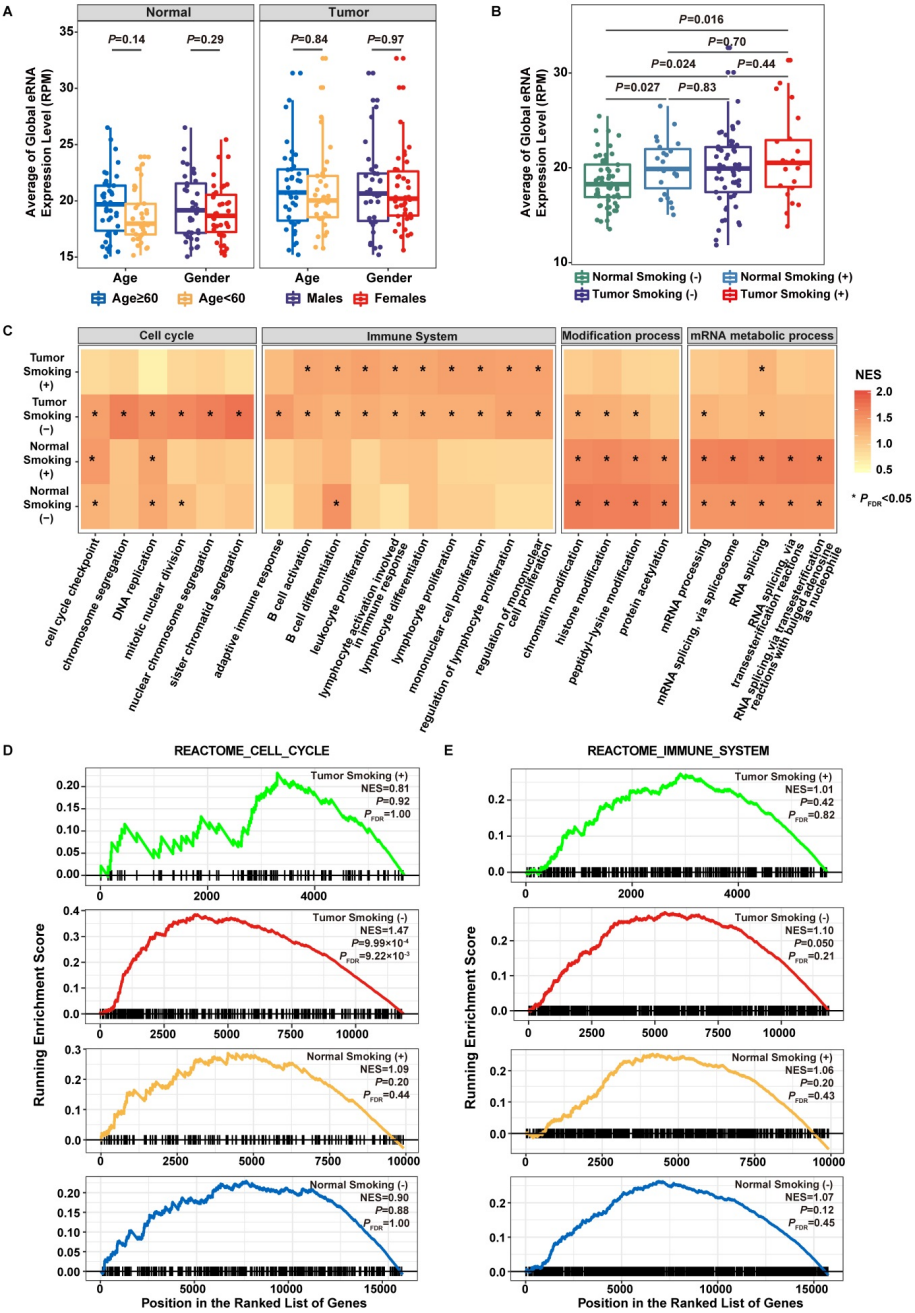
** Functional evaluation of transcribed eRNAs. A.** Association of eRNA expression with age and gender. **B.** Association of eRNA expression with smoking statuses. **C.** Normalized enrichment scores (NES) of the top 20 pathways in lung adenocarcinoma smokers, lung adenocarcinoma non-smokers, adjacent normal smokers, and adjacent normal non-smokers. Color of the bar indicates the normalized enrichment score. Statistical significance levels are depicted by *P_FDR_ < 0.05, **P_FDR_ < 0.01. **D.** Gene Set Enrichment Analysis (GSEA) plot depicts the enrichment of co-expressed protein-coding genes (PCGs) of eRNAs in the Cell Cycle gene set from Reactome pathway dataset. **E.** GSEA plot depicts the enrichment of co-expressed PCGs of eRNAs in the Immune System gene set from Reactome pathway dataset.

When accessing the expression difference of eRNAs among subgroups divided by age, gender, or tobacco smoking history, we found no correlation of eRNA expression with age or gender (**Figure 2A**). However, a significantly elevated eRNA expression was observed in normal tissues of smokers than that in non-smokers (*P =* 0.027) (**Figure 2B**), and the expression level in normal smokers was comparable to that in tumor samples (normal smokers vs. tumor non-smokers: *P =* 0.83; normal smokers vs. tumor smokers: *P =* 0.70) (**Figure 2B**). Differential expression analysis between tumor samples and normal smokers as well as between tumor samples and normal non-smokers also revealed that the expression pattern of eRNAs among normal smokers was more similar to tumor samples than that in normal non-smokers, where significantly fewer differentially expressed eRNAs were observed between normal smokers and tumor samples than that between normal smokers and non-smokers (Fisher's exact test: *P =* 9.75×10^-31^) (**Figure S3**). These findings indicated that processes of tobacco smoking exposure and tumorigenesis could both affect the epigenetic modification of eRNAs.

Co-expression and GSEA analyses were performed to elucidate the biological functions of 3297 eRNAs among four groups of samples (smokers and non-smokers of tumor samples as well as normal adjacent samples) (**Figure 2C**). While eRNA-correlated genes identified in tumor (smoker and non-smoker) samples were primarily involved in immune system-related pathways, *i.e.*, adaptive immune response pathway (tumor smokers: Normalized enrichment score (NES) = 1.15, *P =* 0.091, *P_FDR_*= 0.85; tumor non-smokers: NES = 1.45, *P =* 9.99×10^-4^, *P_FDR_*= 0.042; normal smokers: NES = 0.80, *P =* 0.98, *P_FDR_*= 1.00; normal non-smokers: NES = 0.80, *P =* 0.99, *P_FDR_*= 1.00), eRNA-correlated genes identified in tumor non-smokers were also involved in cell cycle-related pathways, *i.e.*, chromosome segregation pathway (tumor smokers: NES = 0.94, *P =* 0.69, *P_FDR_*= 1.00; tumor non-smokers: NES = 1.66, *P =* 9.99×10^-4^, *P_FDR_*= 0.042; normal smokers: NES = 1.15, *P =* 0.092, *P_FDR_*= 0.62; normal non-smokers: NES = 1.09, *P =* 0.18, *P_FDR_*= 0.54). Genes identified in normal (smoker and non-smoker) samples were primarily involved in modification and mRNA metabolic processes-related pathways. The specific enrichment of eRNA-correlated genes in the cell cycle and immune system-related pathways among tumor samples of non-smokers were replicated when using Reactome and KEGG datasets (**Figure 2D-E**, **Figure S4**).

### Cancer driver genes and clinically-actionable genes are overrepresented in eRNA-correlated genes

**Figure 3:**
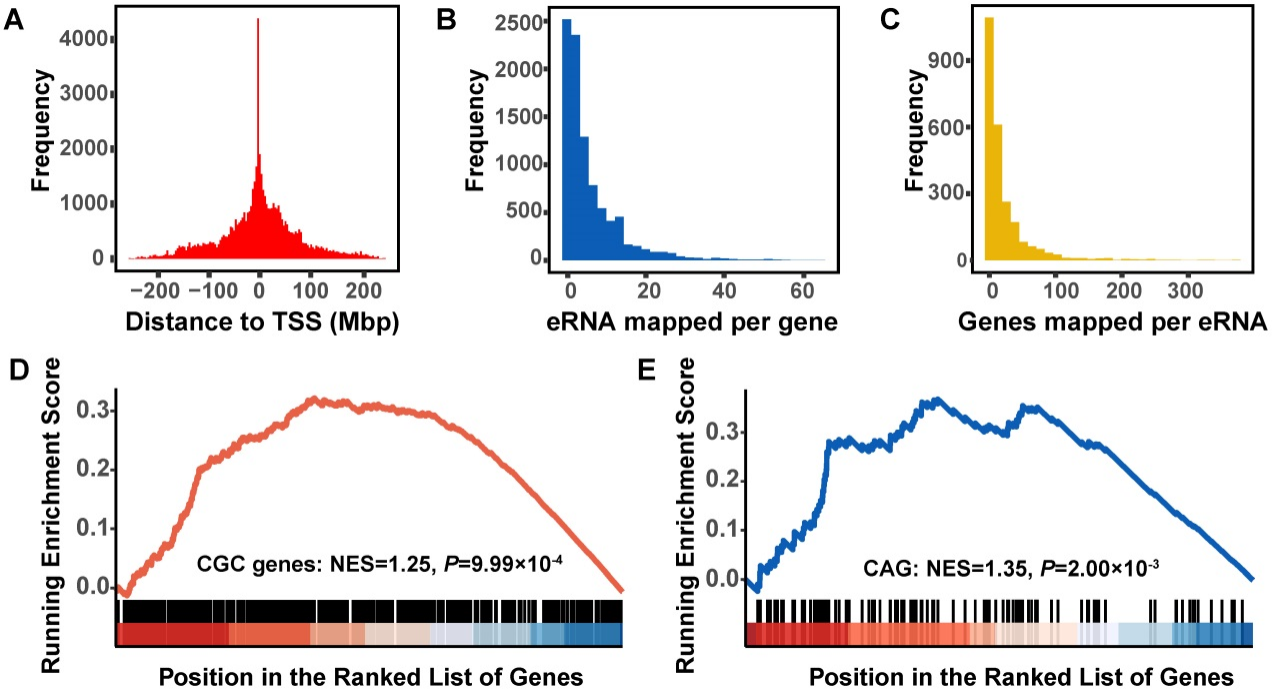
** In silico prediction of functional eRNAs for lung adenocarcinoma. A.** Distribution of the distance of each eRNA to the transcriptional start site (TSS) of the co-expressed protein-coding genes (PCGs). **B.** Distribution of the number of PCGs co-expressed with per eRNA. **C.** Distribution of the number of eRNAs co-expressed with per PCG. **D.** Gene Set Enrichment Analysis (GSEA) plot depicts the enrichment of co-expressed PCGs of eRNAs in Cancer Gene Census (CGC) driver genes. **E.** GSEA plot depicts the enrichment of co-expressed PCGs of eRNAs in clinical actionable genes (CAGs).

To identify eRNAs-correlated genes during lung tumorigenesis, we built a global eRNA-gene regulatory network in tumor samples and identified a total of 14,267 PCGs with significant expression correlations with 3204 eRNAs (absolute value of the correlation coefficient ≥ 0.20, *P_FDR_ <* 0.05), of which 9239 were located in the same chromosome of 2471 co-expressed eRNAs. The histogram of distances between eRNAs and correlated genes decayed sharply with distance (**Figure 3A**), and exhibited a significant enrichment within one Mbp distance (ER = 1.56, Fisher's exact test: *P <* 2.20×10^-16^). Most (52.56%, 4856/9239) PCGs were mapped to less than three different eRNAs (**Figure 3B**), whereas 55.00% (1359/2471) eRNAs were predicted to interact with less than ten correlated PCGs (**Figure 3C**).

We then collected 615 candidate cancer driver genes from Cancer Gene Census (CGC) and 135 clinically-actionable genes (CAGs) of cancer, and identified that 81.46% of CGC genes and 82.96% CAGs were correlated with eRNAs (**Table S7**). These two groups of cancer-related genes were significantly overrepresented among eRNA-correlated genes (CGC: ER = 1.98, *P =* 5.95×10^-12^; CAG: ER = 2.19, *P =* 3.44×10^-4^), and similar results were observed when the GSEA method was applied (CGC: NES = 1.25, *P =* 9.99×10^-4^; CAG: NES = 1.35, *P =* 2.00×10^-3^) (**Figure 3D-E**).

### Functional eRNAs-based clustering is associated with genomic aberrations

**Figure 4:**
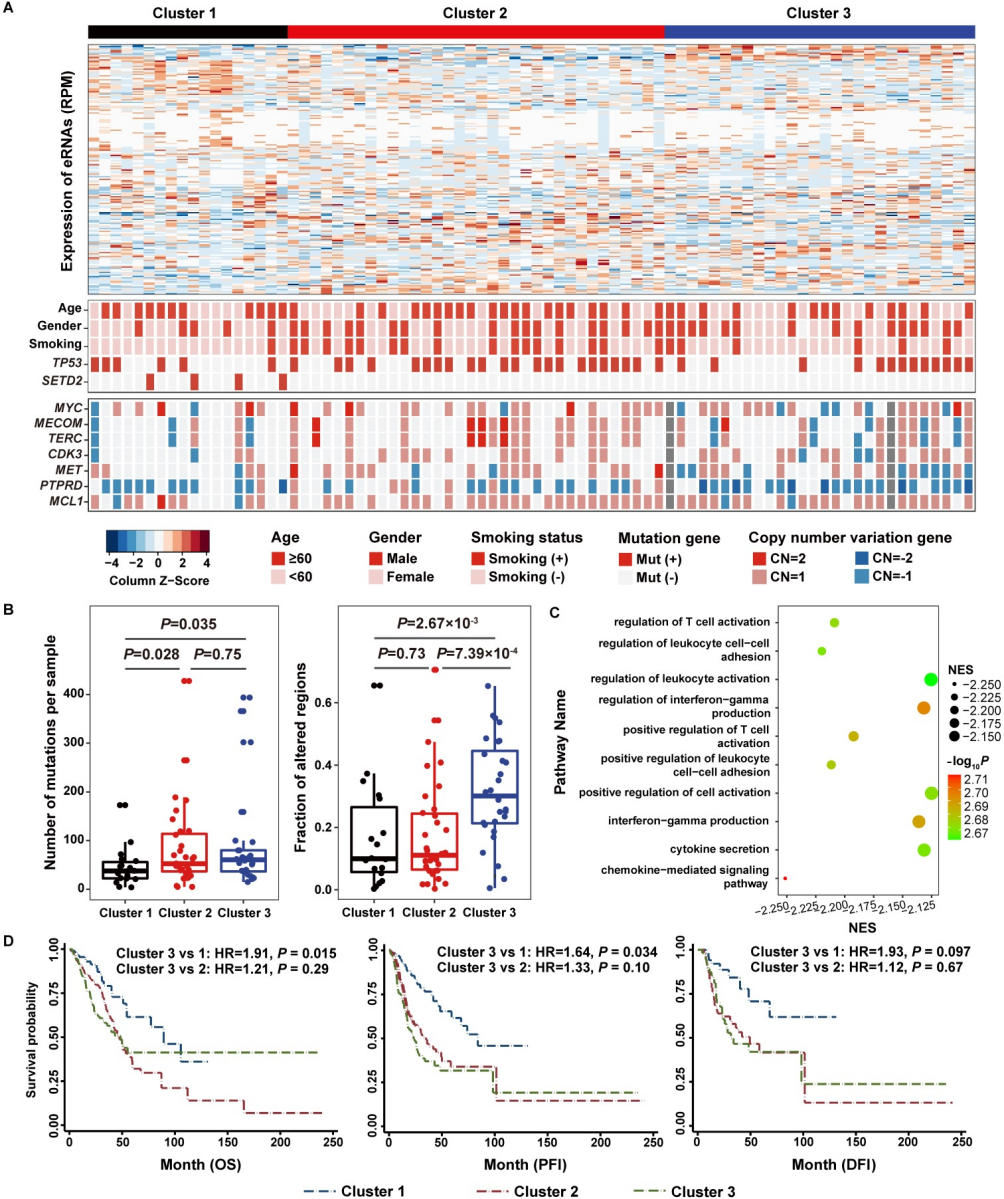
** Consensus Cluster of the expression of 188 functional eRNAs reveals three distinct integrated clusters in lung adenocarcinoma patients. A.** Heatmap representation of 188 functional eRNAs in three clusters. **B.** Number of mutations and fraction of copy number alter genomes per sample in three clusters. **C.** Gene Set Enrichment Analysis (GSEA) of genes differentially expressed in lung adenocarcinoma patients in Cluster 3 from patients in the other two clusters. The *x*-axis and the size of circles indicate the normalized enrichment score (NES) of each pathway. **D.** Survival analysis reveals a prognostic prediction effect of eRNA-based clusters.

Based on the co-expressed eRNA-PCG pairs, we defined 188 (129 upregulated and 59 downregulated) eRNAs with co-expressed upregulated or downregulated PCGs as functional eRNA-PCG pairs for lung adenocarcinoma (**Tables S8-9**) after conducting a series of filtering process as described in the methods section (**Figure 1A**). Consensus clustering analysis based on these 188 eRNAs resulted in three robust clusters (Cluster 1, Cluster 2 and Cluster 3) (**Figure 4A, Figure S5**). Although patients in three clusters had similar expression level, the two most common types of cancer genomic events (somatic mutation and copy number alteration) varied a lot (**Figure 4B**). Patients in Cluster 1 exhibited a normal-like genomic pattern with the lowest level of genomic alterations; however, *SETD2* (one established mutation driver gene of lung adenocarcinoma) mutated only in these patients (*P =* 1.94×10^-3^) (**Figure 4A**). Patients in Cluster 2 were enriched for tobacco smokers (*P =* 2.65×10^-3^) and had a median level of mutation rates and copy number alteration levels (**Figure 4A**, **Figure S6**). As patients in Cluster 3 had the highest level of genomic alterations (**Figure 4B**, **Figure S7**), copy number alterations of many genes were overrepresented, such as *TERC* (*P =* 0.015), *PTPRD* (*P =* 0.012), and *MYC* (*P =* 0.039) (**Figure 4A**). Co-expression analysis and GSEA also revealed that PCGs co-expressed with eRNAs in these three groups of patients were involved in different biological pathways (**Table S10**).

The same analyses were conducted in 481 TCGA lung adenocarcinoma samples, and 80.85% (152/188) of the functional eRNAs and corresponding eRNA-PCG pairs were validated. The eRNA-based clustering also grouped TCGA patients into three clusters with different somatic mutation loads and copy number alteration levels (**Figure S8A-B**). Strong concordances were observed when comparing our eRNA-based clusters to the multiomic iCluster scheme reported in a previous published study 7 using RNA-seq, microRNA-seq, DNA methylation, reverse-phase protein array, and DNA copy number data. When compared to mRNA-based subtypes (**Figure S8A**), we identified that patients in Cluster 3 were enriched in the proximal-proliferative transcriptional subtype (*P =* 9.14×10^-18^) which was characterized by a high level of chromosome loss 7, and patients in Cluster 2 were enriched in the terminal respiratory unit (TRU) group (*P =* 0.043). In consistent with previous report that TRU group was characterized by a high level of *EGFR* mutations 7, we also observed significantly more *EGFR* mutations in eRNA-based Cluster 2 patients from the TCGA dataset (*P =* 1.26×10^-6^) (**Figure S9**). When compared to methylation-based subtypes (**Figure S8A**), Cluster 2 patients were overrepresented in the CpG island methylator phenotype (CIMP)-high and intermediate groups (*P =* 7.04×10^-5^). While CIMP-high and intermediate groups were characterized by a high *SETD2* mutation rate 7, we also found more *SETD2* mutations in eRNA-based Cluster 2 patients, although didn't reach the significance level (*P =* 0.065).

### Specific eRNA-based cluster is predictive of poor clinical outcomes

As patients in Cluster 3 exhibited the highest genomic instability level, we identified that genes downregulated in this group of patients compared to the other two groups of patients were primarily involved in immune related pathways from both NJLCC (**Figure 4C, Tables S11**) and TCGA (**Tables S12**) projects. In addition, proportions of four types of immune cells were attenuated in Cluster 3 patients compared to patients in other two clusters from NJLCC project (CD4 T cell: *P =* 1.62×10^-3^; Neutrophil: *P =* 2.02×10^-4^; Macrophage: *P =* 6.73×10^-3^; Dendritic cell: *P =* 1.03×10^-3^), suggesting that the immune system may be affected (**Figure S10**). Cluster 3 patients in TCGA further confirmed the results for proportions of these four immune cell types (**Figure S10**). Further co-expression analysis between 188 functional eRNAs and 40 immune checkpoints collected from Ruppin et.al 43 also revealed that significantly less positive (Cluster 3 *vs.* Clusters 1 & 2: ER = 0.48, *P =* 7.34×10^-25^) and negative (Cluster 3 *vs.* Clusters 1 & 2: ER = 0.73, *P =* 9.76×10^-4^) immune checkpoint-eRNA pairs were observed among Cluster 3 patients than that in Cluster 1 and 2 patients (**Table S13**).

Then, we accessed the clinical outcomes of patients in Custer 3, and identified that this group of patients had a relatively worse survival status compared to patients in Cluster 1 with OS (Cluster 3 *vs.* 1: HR = 1.91, *P =* 0.015; Cluster 3 *vs.* 2: HR = 1.21, *P =* 0.29) and PFI (Cluster 3 *vs.* 1: HR = 1.64, *P =* 0.034; Cluster 3 *vs.* 2: HR = 1.33, *P =* 0.10) as the endpoints, but not for DFI (Cluster 3 *vs.* 1: HR = 1.93, *P =* 0.097; Cluster 3 *vs.* 2: HR = 1.12, *P =* 0.67) (**Figure 4D**).

### Copy number amplification activates eRNA expression in lung adenocarcinoma

**Figure 5:**
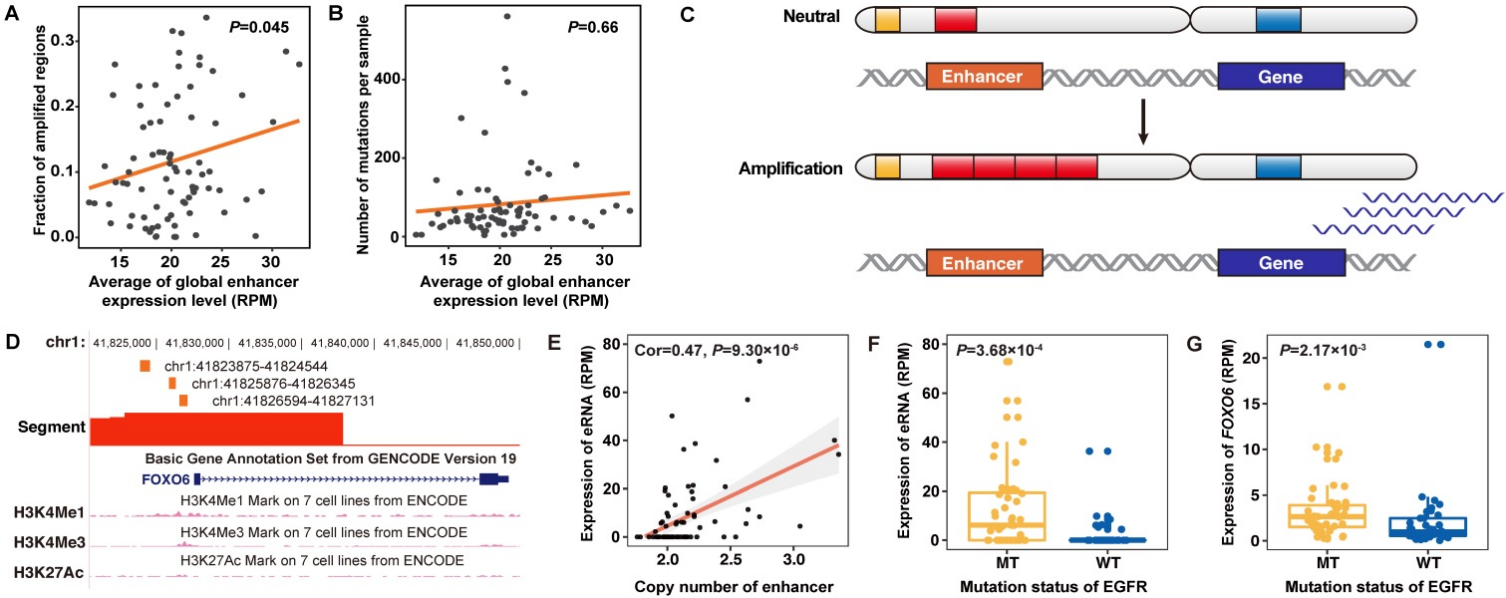
** Copy number amplification related eRNAs and correlated genes in lung adenocarcinoma. A.** Correlation between the global expression of eRNAs in lung adenocarcinoma samples and the genomic copy number amplification level. **B.** Correlation between the global expression of eRNAs in lung adenocarcinoma samples and the genomic mutation burden of non-silent mutations. **C.** Models of the definition of copy number amplification-related eRNAs. Red bars in the chromosome indicate the copy number level of eRNA regions and blue bars in the chromosome indicate the copy number level of eRNA-related genes. Amplification of the copy number of eRNA regions will lead to upregulated expression of eRNA-related genes. **D.** Genomic annotation of the *FOXO6* region. The orange bar indicates the genomic location and length of three *FOXO6*-eRNAs, and the red bar indicates the segment of the copy number of specific genomic regions. **E.** The expression of *FOXO6*-eRNA was significantly associated with the copy number level of the eRNA region. **F.** The expression of *FOXO6*-eRNA was significantly higher in samples with *EGFR* mutations. **G.** The expression of *FOXO6* was significantly higher in samples with *EGFR* mutations.

When accessing the effect of genomic alterations on global-eRNA expression, we observed a positive association with fractions of amplified genome (Cor = 0.22, *P =* 0.045) (**Figure 5A**), where no significant association was found for somatic mutation rates (Cor = -0.05, *P =* 0.66) (**Figure 5B**). By integrating the copy number information (**Figure 5C**), we identified that four of above defined 129 upregulated eRNAs targeting *FOXO6*, *TERT* and *PAX9* were activated by CNA in lung adenocarcinoma samples.

As a classic lung cancer related gene, the expression of two-candidate functional eRNAs in the *TERT* region were highly correlated with their copy number levels (**Figure S11A-B**). Other six eRNAs in this region also showed elevated expression in tumor samples (**Figure S11C**). Another interesting finding was the identification of *FOXO6* (**Figure 5D-E**) as a novel driver gene for lung adenocarcinoma. We observed significantly elevated expression of *FOXO6*-eRNA and *FOXO6* in *EGFR* mutated samples in both NJLCC (*FOXO6*-eRNA: *P =* 3.68×10^-4^, *FOXO6*: *P =* 2.17×10^-3^) (**Figure 5F-G**) and TCGA samples (*FOXO6*-eRNA: *P =* 1.66×10^-10^, *FOXO6*: *P =* 6.96×10^-8^) (**Figure S12**), suggesting that the activation of *FOXO6*-eRNA may be *EGFR*-dependent. Further qPCR also revealed that the expression of one *FOXO6*-eRNA was significantly higher in PC9 cell line (*EGFR* mut-type) than that in other two *EGFR* wide-type lung adenocarcinoma cell lines (A549 and NCI-H1299) (**Figure S13**).

## Discussion

Here we provided an initial characterization of eRNA landscape in 80 Chinese lung adenocarcinoma patients, and observed an elevated global-eRNA expression among tumor samples compared to normal samples, which predominantly regulate cell cycle and immune related genes. We also defined 188 functional eRNAs and the correlated target genes were overrepresented in cancer driver genes (ER = 1.98, *P =* 5.95×10^-12^) and clinically-actionable genes (ER = 2.19, *P =* 3.44×10^-4^). Consensus clustering of these 188 eRNAs identified a novel molecular subtype with immune deficiency and a high-level of genomic alterations, which was associated with the poor clinical outcomes. Taken together, our findings present a comprehensive description of eRNAs in lung adenocarcinoma, which provide a new biological dimension complementary to other genomic features in understanding the molecular mechanisms underlying lung carcinogenesis. The clinical utility of eRNA-based molecular subtypes also provides implications for the treatment of lung adenocarcinoma.

Uncontrolled cell proliferation and tumor-promoting inflammation are two hallmarks of cancer 44, which enable cancer cells acquiring genomic alterations and lead to genome instability 45. In this study, we identified that eRNAs expressed in lung adenocarcinoma (smoker and non-smoker) samples typically dysregulate genes in cell cycle and immune system pathways, where cell cycle-specific regulation ensures the inheritance of reversible epigenetic markers from generation to generation 46. In addition, although tobacco exposure also modifies epigenetic alterations in normal cells 47, 48 by affecting genes involved in maintaining normal cellular structure 49, we proposed that the effect may be greatly attenuated in cancer cells because these cells are highly disordered 50. These findings provided us a better understanding of the different epigenetic regulation mechanisms underlying both the smoking process where normal structure is damaged, and the tumorigenesis process where highly disordered cancer cells are often more unstable.

Previous studies have provided numerous insights into the effect of somatic mutations and copy-number alterations in modifying gene expression during tumorigenesis 51, 52. In this study, we proposed that focal genomic amplification is more likely to activate eRNA expression during cancer development than somatic mutations, which was consistent with previous findings 53. Of the three amplification-related driver genes implicated in this study, *TERT*, the gene encodes human telomere reverse transcriptase that maintain telomere ends 54, is a classic predisposition gene for lung cancer 55, 56. Here, we identified that the highly upregulated eRNAs upstream of *TERT* may contribute to lung cancer development by upregulating the expression of *TERT*. Another interesting result is the identification of *FOXO6*, a member of Forkhead transcription factors 57, as a novel driver gene for lung adenocarcinoma. *FOXO*6 expression was upregulated in lung adenocarcinoma, which was predominantly attributed by the CNA of *FOXO6*-eRNA. Although the tumor-promoting role of *FOXO6* in adenocarcinoma has not been reported, this TF was previously found to contribute to the resistance of erlotinib treatment in *EGFR*-mutant lung cancers by inducing the expression of SOX2 58. Thus, in addition to EGFR-FOXO6-SOX2 feedback loop, the expression of *FOXO6* can also be regulated by *FOXO6*-eRNA, which provided novel implications for the targeted therapy of FOXO6-related erlotinib resistance in lung cancer patients.

With the progression of cancer sequencing studies, molecular-targeted therapies are increasingly used as an alternative to chemotherapy 59, 60, which requires the identification of candidate targets with key roles in the growth and survival of cancer cells. A recent study investigated the clinical feasibility of eRNA-targeted therapy and confirmed the therapeutic liability of NET1e 29. Here, we also found an enrichment of CAGs collected from Tumor Alterations Relevant for Genomics-driven Therapy (TARGET) database 61 among eRNA-correlated genes, which provided additional evidence for the clinical potential of eRNAs in lung adenocarcinoma. Moreover, our eRNA-specific clustering enabled the discovery of a novel subtype with immune deficiency and correlates with a malignant progression state. As this group of samples is enriched for high somatic mutation and copy number alteration loads, they may have a better response to immune checkpoint inhibitors 62, 63. In addition, we identified that patients in the low-mutation group specifically carry *SETD2* mutations. *SETD2* is a gene encodes the histone H3K36 methyltransferase 64 and loss of *STED2* could dysregulate methyltransferase activity, which was therapeutically manipulate 65. Generally, targeted therapy is not applicable for patients with low mutation rates 66; but our study proposed that patients with *SETD*2 mutations may serve as a potential cohort for epigenetic therapy 65.

Although this study provided novel information and intriguing insights into understanding eRNAs underlying the development of lung cancer, our findings should be interpreted in the context of some limitations. First, the limited sample size impacts the overall statistical power of our study. Second, the structure of our defined eRNAs is uncertain, given that we do not have long-range chromosome interaction data, such as Chip-seq or Hi-C. Third, since only limited lung-specific enhancer annotation datasets are available, lung-related eRNAs may be underestimated. Finally, because eRNAs usually expressed at a very low level and may get degraded quickly, GRO-seq is a commonly used technology for the identification of active enhancers 26, 67. However, RNA-seq used in this study can also be utilized for eRNA quantification 28, 29. When compared to the nascent enhancers detected with GRO-seq of a lung adenocarcinoma cell line (A549), most (68.6%, 2262/3297) of our defined expressed eRNAs in lung adenocarcinomas could be identified (**Table S14**). Additionally, qRT-PCR also validated the expression of our defined eRNAs in corresponding lung adenocarcinoma samples (**Figure S14**), suggesting a high accuracy of our method used for eRNA identification.

In summary, we provided a global view of active eRNAs in lung adenocarcinoma and proposed that the transcriptional profile of eRNAs represents a novel biological dimension complementary to other genomic features. These findings are of great importance as it not only provides a better understanding of the mechanisms underlying lung carcinogenesis, but also provides clinical implications for the treatment of lung adenocarcinoma.

## Supplementary Material

Supplementary figures and tables.

Supplementary tables.

## Abbreviations

CAG: clinically-actionable gene
CI: confidence interval
CIMP: CpG island methylator phenotype
CNA: copy number amplification
DFI: disease-free interval
ENCODE: the Encyclopedia of DNA Elements
eRNAs: enhancer RNA
FPKM: fragments per kilobase of exon per million reads mapped
HR: hazard ratio
NES: Normalized enrichment score
NJLCC: Nanjing Lung Cancer Cohort
OS: overall survival
PCG: protein-coding gene
PFI: progression-free interval
RPM: reads per million mapped reads
TCGA: The Cancer Genome Atlas
TF: transcriptional factor
TPM: Transcripts Per Kilobase of exon model per Million mapped reads
TRU: terminal respiratory unit
WES: whole-exome sequencing
WGS: whole-genome sequencing
