# Supplementary material for: Comprehensive characterization of functional eRNAs in lung adenocarcinoma reveals novel regulators and a prognosis-related molecular subtype
Source: Theranostics. 2020 Sep 14;10(24):11264–77. doi: 10.7150/thno.47039 (PMC7532687; doi:10.7150/thno.47039)
Supplement: Supplementary file 1 — Supplementary figures and tables. [file thnov10p11264s1.pdf]

Supplementary Materials

Qin N, Ma Z, Wang C, et al. Comprehensive characterization of functional eRNAs in lung adenocarcinoma reveals novel regulators and a prognosis-related molecular subtype.

Supplementary Figures

Supplementary Figure 1. Schematic workflow for the eRNA identification process.

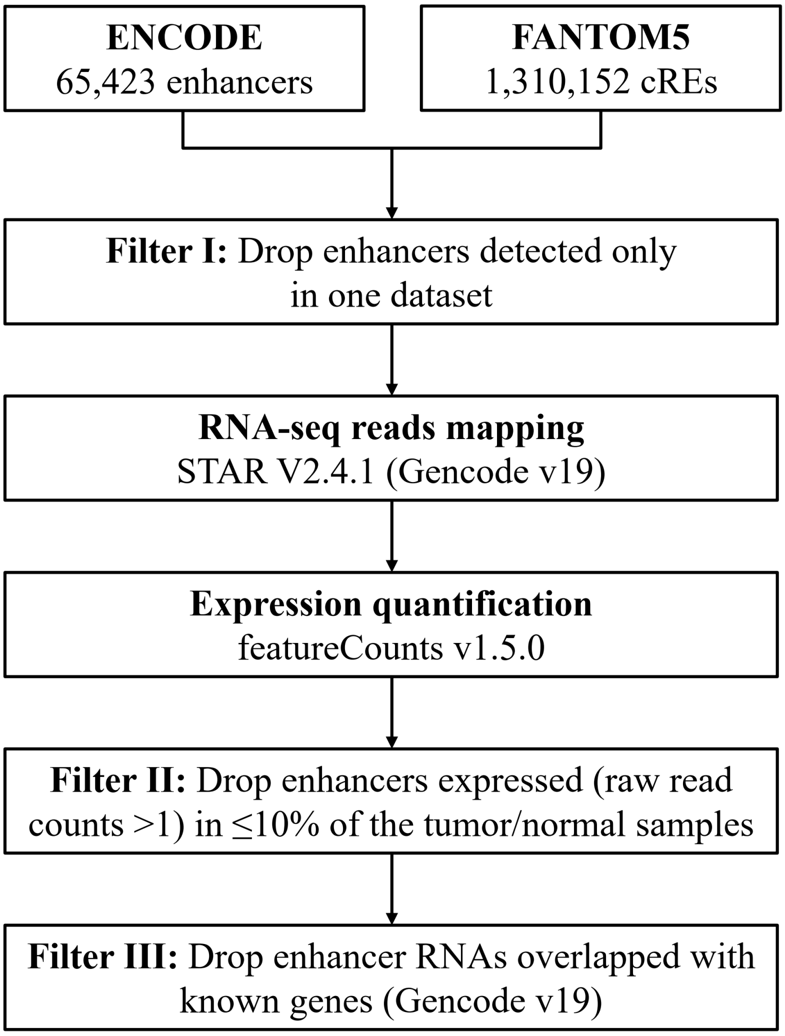

Supplementary Figure 2. Expression correlations of transcribed eRNAs with EP300 and POLR2A in lung adenocarcinoma samples.

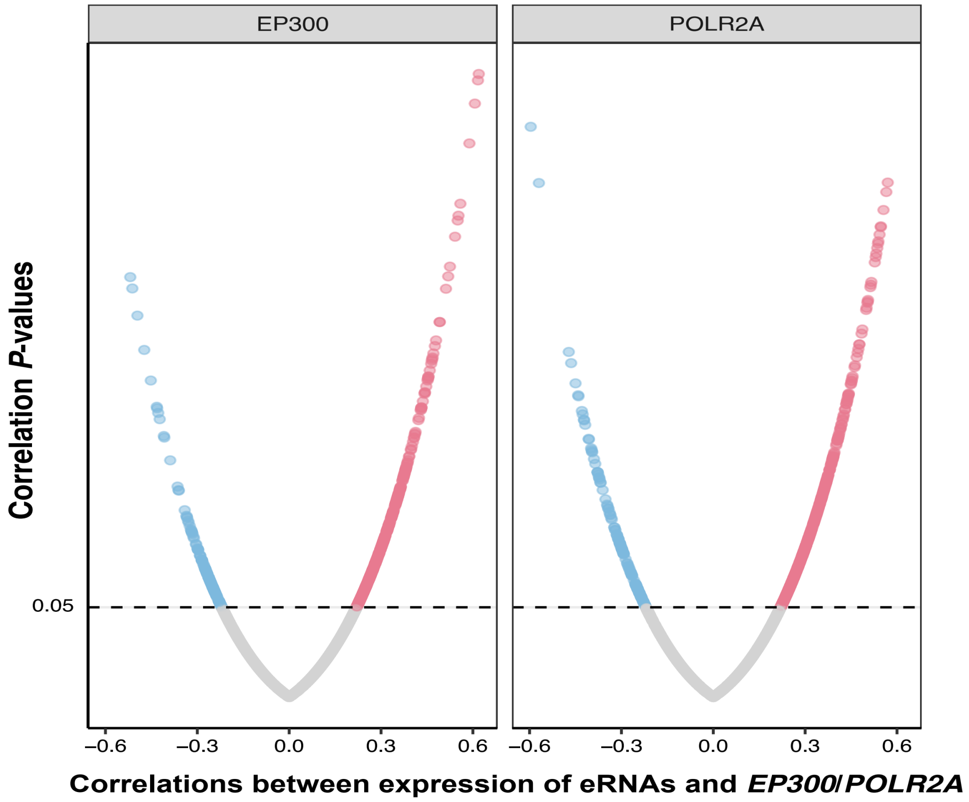

Supplementary Figure 3. Number of differentially expressed eRNAs between tumor samples and normal samples with different smoking status.

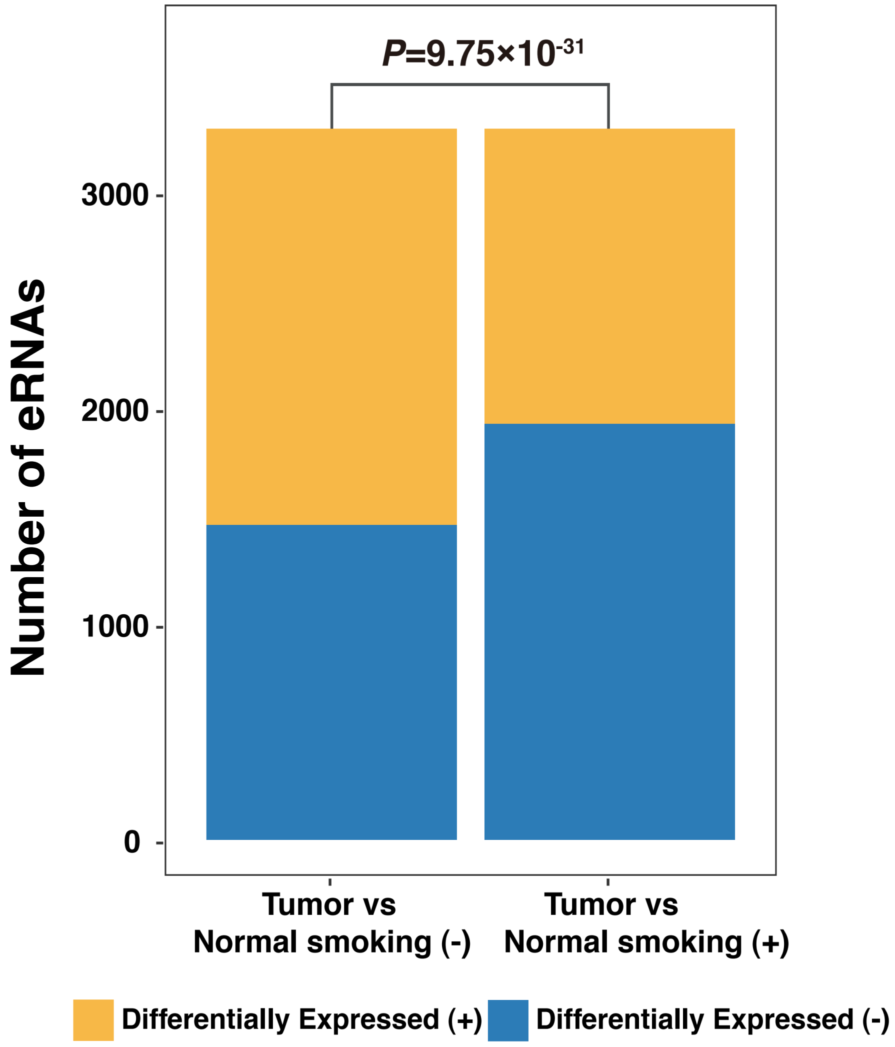

Supplementary Figure 4. GSEA plot depicts the enrichment of co-expressed protein-coding genes of eRNAs in KEGG Cell Cycle and Huamn T-cell leukemia virus 1 infection gene sets.

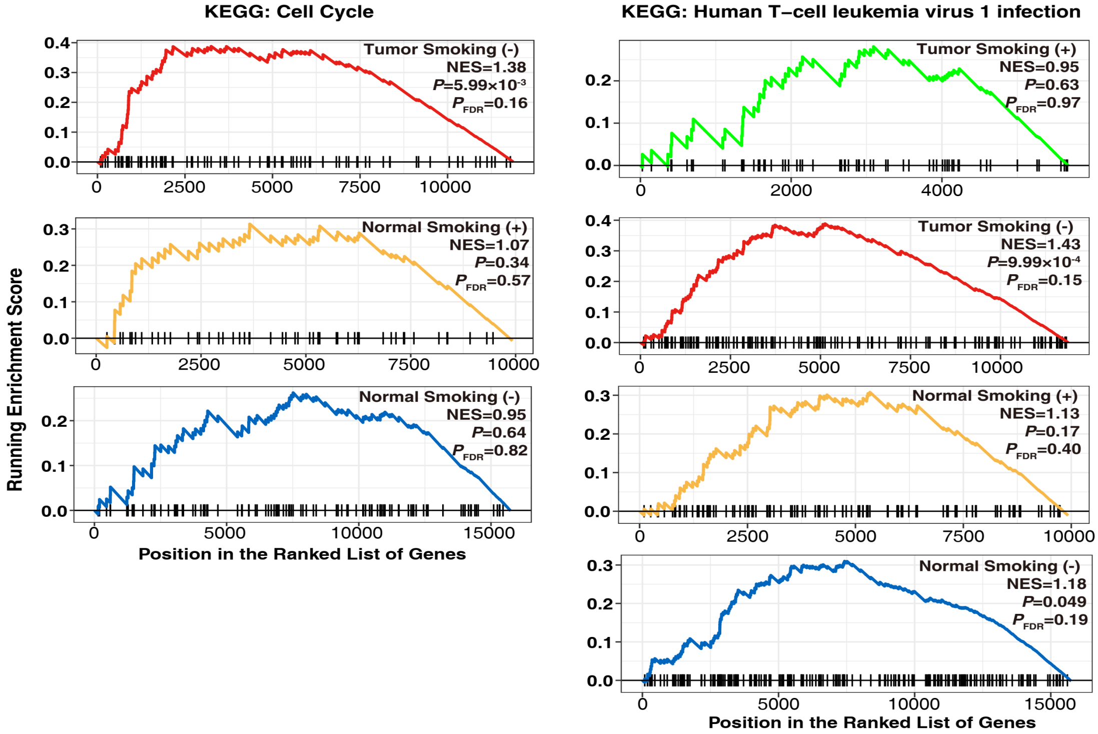

Supplementary Figure 5. Unsupervised consensus clustering of expression of 188 functional eRNAs.

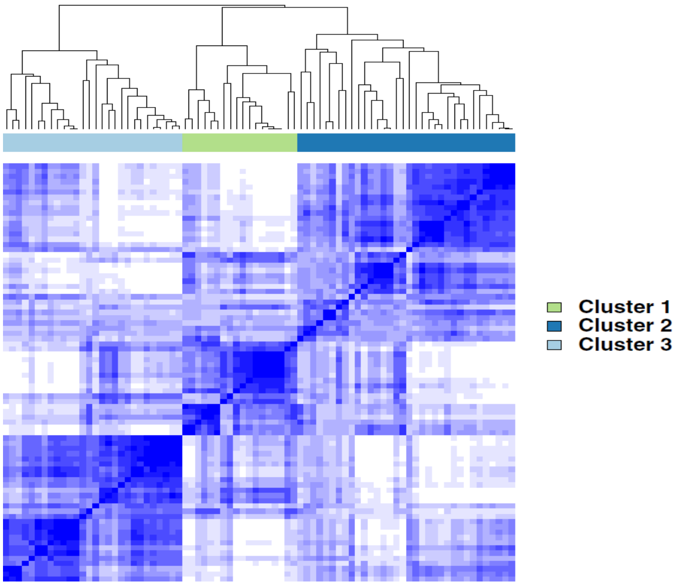

Supplementary Figure 6. Proportions of patients with smoking history in three clusters of NJLCC lung adenocarcinomas.

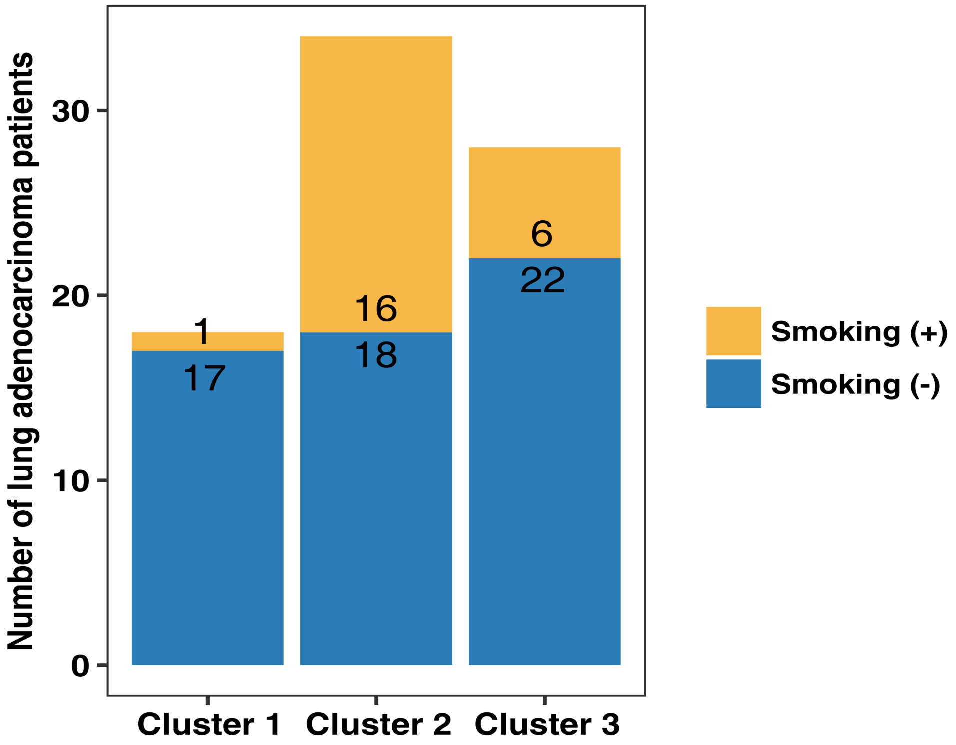

Supplementary Figure 7. Fraction of amplified or deleted regions per sample in three clusters of NJLCC lung adenocarcinomas.

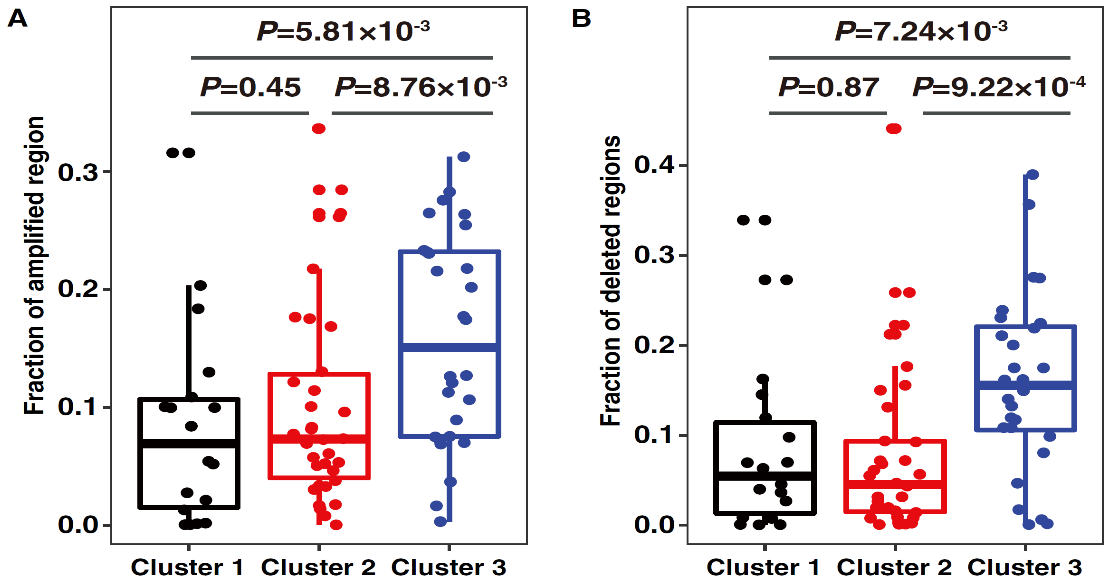

Supplementary Figure 8. Integrative analysis of candidate functional eRNAs with ConsensusCluster in TCGA lung adenocarcinoma patients

A. Heatmap representation of functional eRNAs in three clusters.

B. Number of mutations and fraction of copy number alter genomes per sample in three clusters.

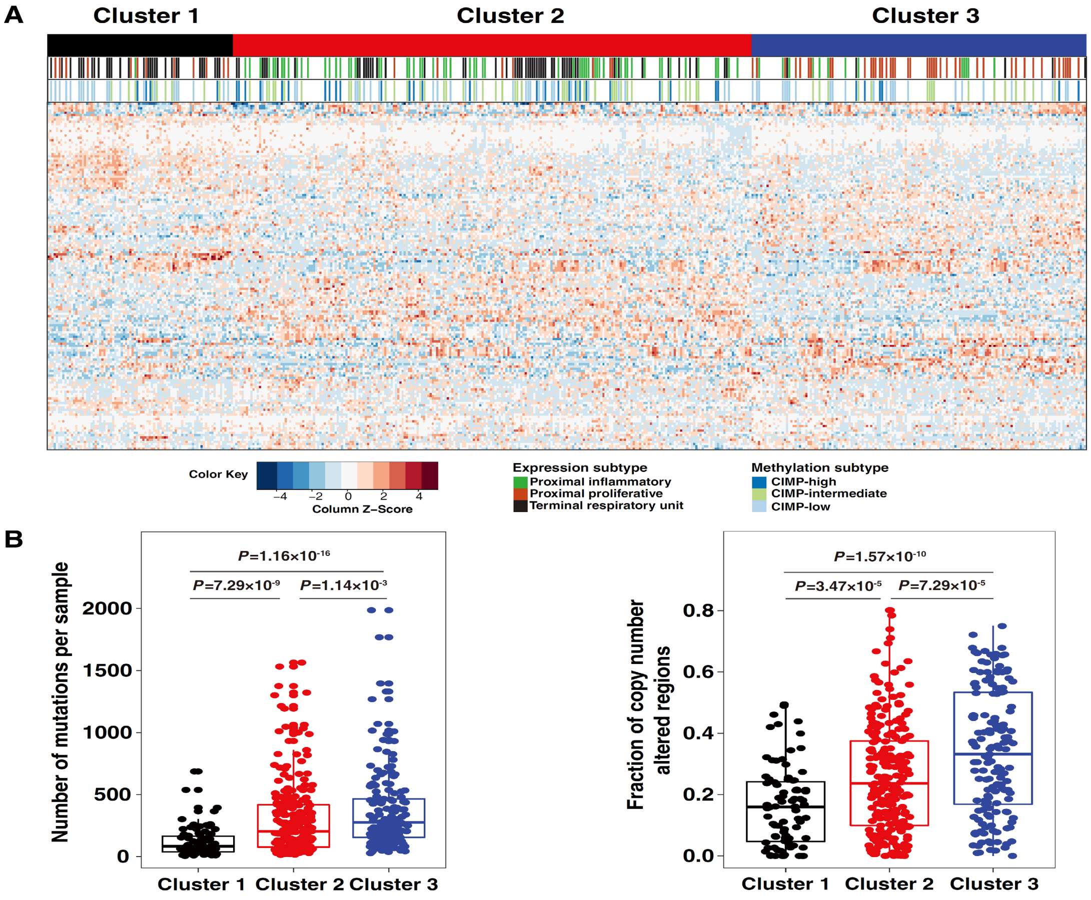

Supplementary Figure 9. Proportions of patients carry EGFR somatic mutations among Cluster 2 and other two clusters in TCGA data.

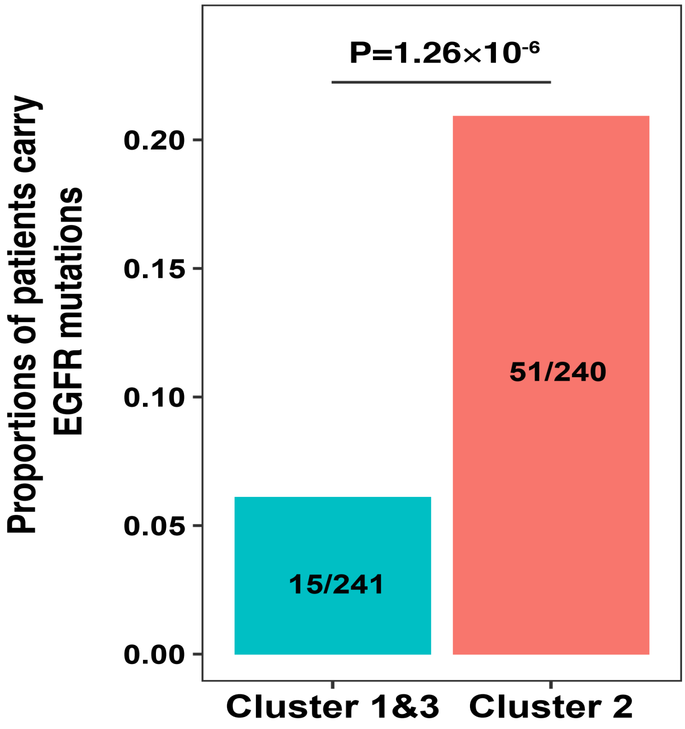

Supplementary Figure 10. Estimated immune cell proportions in NJLCC (A) and TCGA (B) adenocarcinoma samples.

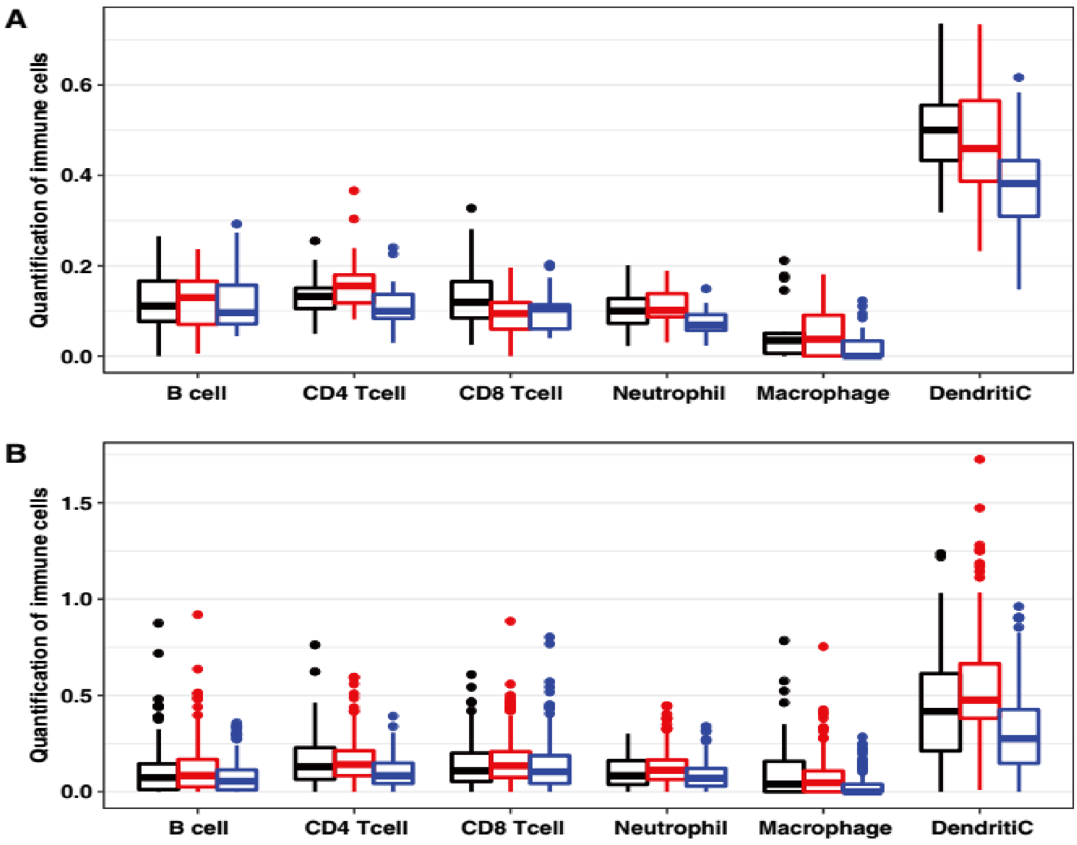

Supplementary Figure 11. Detailed information for the *TERT* region

A-B. Expression of the *TERT*-eRNA were significantly associated with the copy number level of the eRNA

C. Expression of transcribed eRNAs in the *TERT* region were significantly higher in lung adenocarcinoma samples than adjacent normal samples

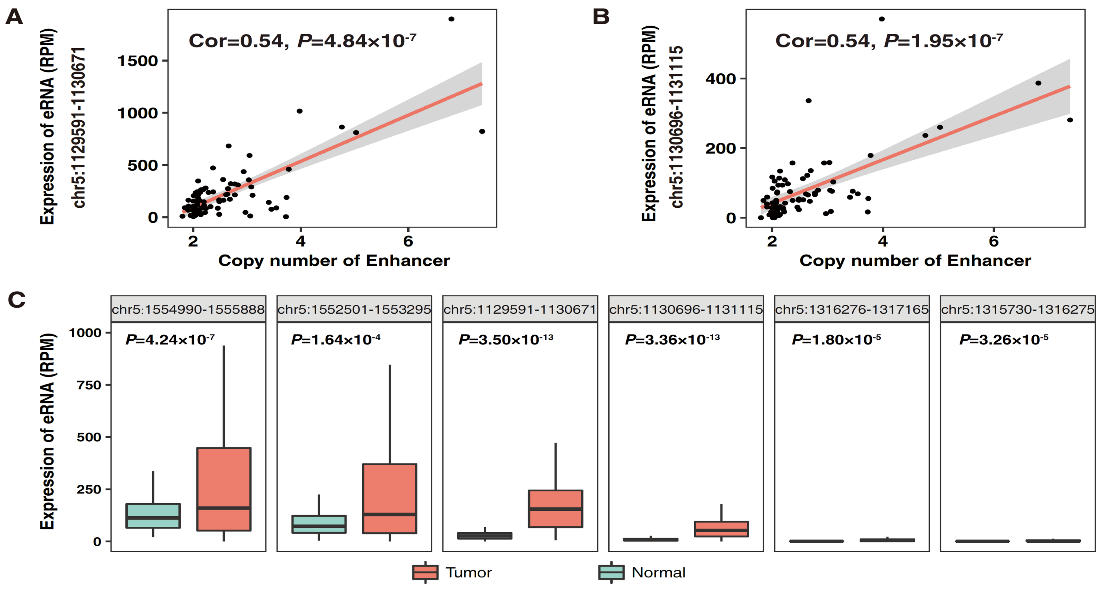

Supplementary Figure 12. Association of *EGFR* mutations with *FOXO6* and *FOXO6*-eRNA expression.

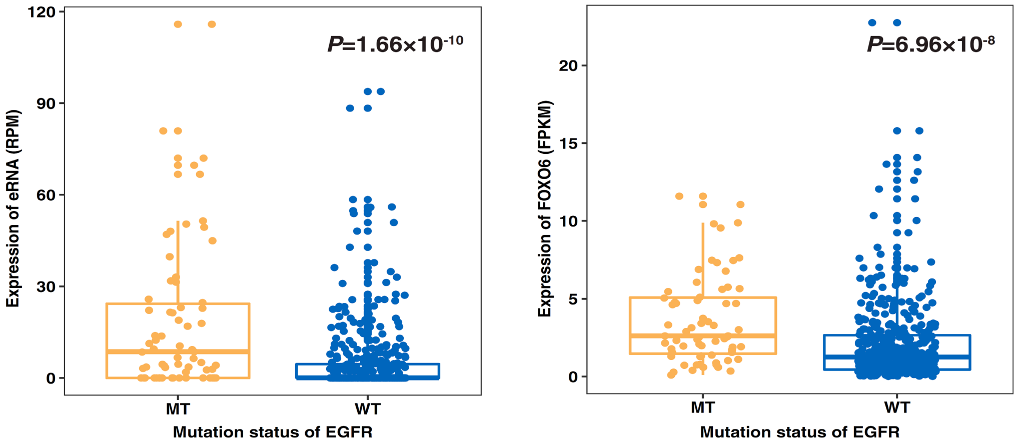

Supplementary Figure 13. The Quantitative Real-time PCR (qRT-PCR) of three *FOXO6*-eRNAs in lung adenocarcinoma cell lines.

EGFR mut-type cell line: PC9, *EGFR* wide-type cell lines: A549 and NCI-H1299.

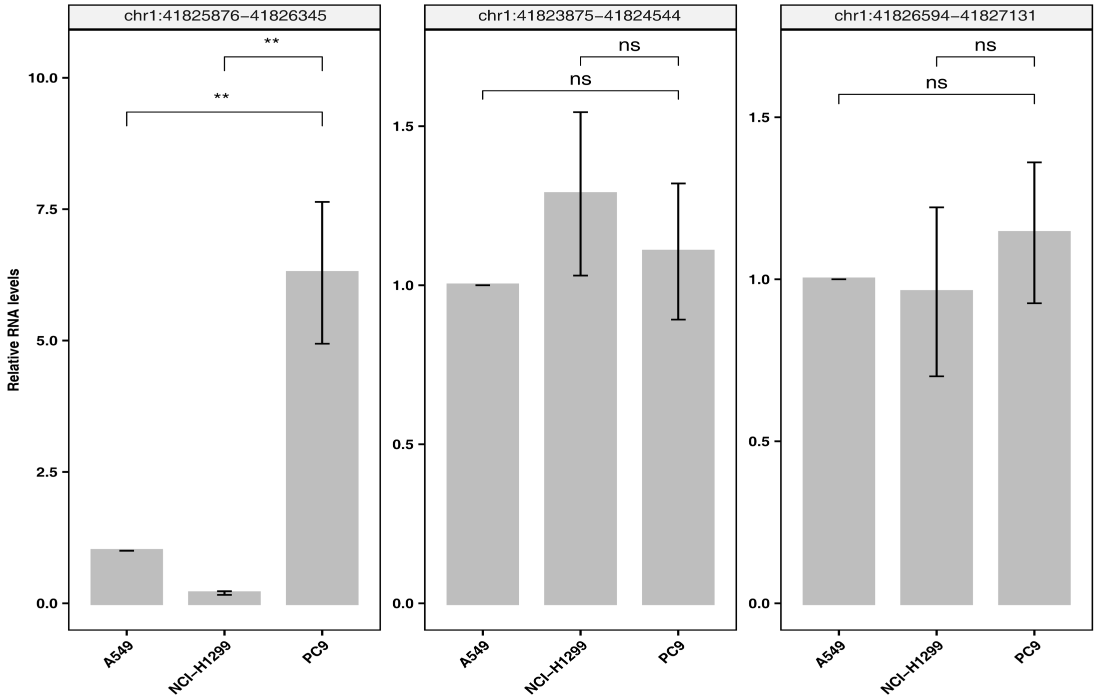

Supplementary Figure 14. The Quantitative Real-time PCR (qRT-PCR) of ten eRNAs in lung adenocarcinoma samples.

Cycle threshold [Ct] value below 30 is defined as expressed.

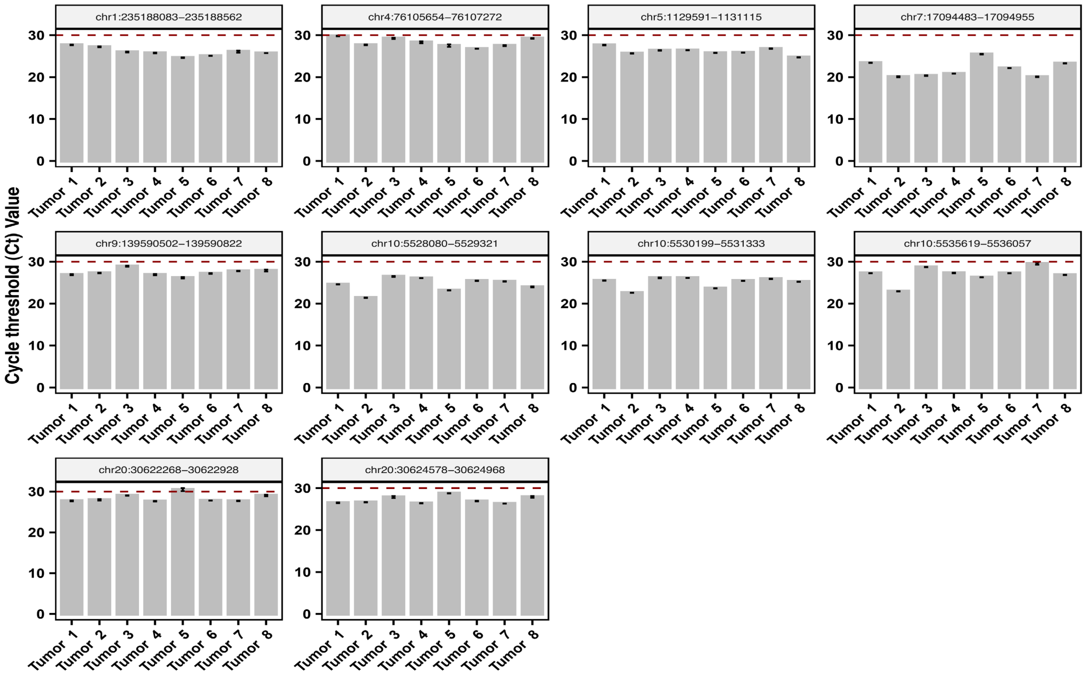

Supplementary Table 1. General description of lung adenocarcinoma samples used in this study.

	NJLCC n=80	TCGA n=481
Age (median; range)	61.6 (34.0-83.0)	67.0 (37.0-88.0)
Gender		
Male	38 (47.5)	220 (45.7)
Female	42 (52.5)	261 (54.3)
Smoking History		
Ever smoker	23 (28.8)	409 (85.0)
Lifelong non-smoker	57 (71.2)	72 (15.0)

Abbreviations: NJLCC, Nanjing Lung Cancer Cohort; TCGA, The Cancer Genome Atlas.

Supplementary Table 2. Sequenced reads for 80 tumor-adjacent paired lung adencocarcinoma patients from NJLCC study.

Patient ID	Total_ReadCounts_Tumor (million)	Total_ReadCounts_Normal (million)
Patient 1	71.86	78.96
Patient 2	62.49	71.80
Patient 3	73.44	73.64
Patient 4	73.80	89.64
Patient 5	72.43	67.17
Patient 6	64.36	90.05
Patient 7	90.62	85.57
Patient 8	89.12	72.01
Patient 9	73.00	84.81
Patient 10	87.74	90.91
Patient 11	72.29	63.18
Patient 12	109.35	90.32
Patient 13	87.81	96.69
Patient 14	64.72	89.96
Patient 15	62.23	64.67
Patient 16	86.93	85.46
Patient 17	75.61	87.60
Patient 18	65.16	74.52
Patient 19	89.00	76.23
Patient 20	96.70	90.55
Patient 21	100.75	98.01
Patient 22	54.65	73.40
Patient 23	61.72	57.88
Patient 24	95.06	59.30
Patient 25	46.45	42.49
Patient 26	64.81	84.76
Patient 27	36.53	62.29
Patient 28	48.05	54.22
Patient 29	83.80	62.66
Patient 30	59.81	57.76
Patient 31	37.88	85.03
Patient 32	47.31	39.42
Patient 33	46.00	55.51
Patient 34	40.11	41.57
Patient 35	65.45	52.14
Patient 36	50.59	45.07
Patient 37	62.02	57.63
Patient 38	59.43	52.86

Patient 39	60.73	57.44
Patient 40	70.97	76.33
Patient 41	67.90	67.81
Patient 42	87.18	78.48
Patient 43	74.40	83.27
Patient 44	80.04	89.42
Patient 45	63.53	61.03
Patient 46	64.22	66.32
Patient 47	60.92	61.69
Patient 48	64.39	70.56
Patient 49	66.53	69.01
Patient 50	67.00	68.78
Patient 51	69.88	75.15
Patient 52	74.70	78.03
Patient 53	79.19	62.10
Patient 54	57.81	76.56
Patient 55	67.35	74.62
Patient 56	84.55	78.68
Patient 57	75.37	77.10
Patient 58	77.40	56.98
Patient 59	60.82	66.73
Patient 60	63.38	71.78
Patient 61	73.47	77.22
Patient 62	74.93	66.17
Patient 63	41.89	54.35
Patient 64	86.21	47.69
Patient 65	69.40	90.26
Patient 66	77.82	66.69
Patient 67	69.04	56.33
Patient 68	45.94	68.27
Patient 69	65.40	59.79
Patient 70	54.16	78.83
Patient 71	97.04	68.66
Patient 72	61.16	65.60
Patient 73	73.38	64.46
Patient 74	72.48	64.97
Patient 75	64.85	72.37
Patient 76	71.55	69.50
Patient 77	58.84	64.19
Patient 78	68.26	62.81
Patient 79	150.39	156.34
Patient 80	193.43	151.20

Supplementary Table 4. The sequence of primers used for eRNA quantification.

Prime ID	Sequence
chr1:41823875-41824544 F	TGCCTGCATGTGGAAAAACG
chr1:41823875-41824544 R	CCTACCACGCCCACATTCAT
chr1:41825876-41826345 F	ACGGAGCTTTGAAGAGTGGG
chr1:41825876-41826345 R	CAGGCTGCTGAGCTAGGTTT
chr1:41826594-41827131 F	TCCTCTCCGGAGTAGAAGGC
chr1:41826594-41827131 R	TAACACTCCCATTGGTCCGC
chr1:235188083-235188562 F	GTGCTGTTCCACACTGGAGA
chr1:235188083-235188562 R	GTACCCGGGCAATAGGGAAG
chr4:76105654-76107272 F	AGGTTGAAATCCCTGCCTTCC
chr4:76105654-76107272 R	GGTCTAGGGAGAGTTATGGCT
chr5:1129591-1131115 F	TGTCTCCGCTCAACACCCAG
chr5:1129591-1131115 R	CATTCAGCCTCTCTTCCTCCCAC
chr7:17094483-17094955 F	GAGTCCACTCACCTCCACCA
chr7:17094483-17094955 R	CGTGTGTTGAGACCTTCCTCT
chr9:139590502-139590822 F	CATGTGCCCTGAGTGTCTCC
chr9:139590502-139590822 R	CTTGGTGGGAGTGTTGGTGT
chr10:5528080-5529321 F	TTACAACCCAGTCCAGCAGC
chr10:5528080-5529321 R	TCCATGGGCACAAGTGACAG
chr10:5530199-5531333 F	CTTAATCTTGGCCGCACAGC
chr10:5530199-5531333 R	TGGAGAATGTGCCGTGTGAG
chr10:5535619-5536057 F	GGGAGGAGTGGCCCTGTTTA
chr10:5535619-5536057 R	TCCTGTACCTCCAGGGTGTAG
chr20:30622268-30622928 F	GGGATAGCTGGTTCCCAAGG
chr20:30622268-30622928 R	TTGGGAGAGGCGTCCATCTA
chr20:30624578-30624968 F	GCAACGTGCAGAAAATGATGC
chr20:30624578-30624968 R	CAAACGCACTCCCCCTTTG

Supplementary Table 5. Enrichment analysis of expressed eRNAs in transcriptional factor binding sites.

Cell line	Number of transcribed enhancers in TFBS	Number of untranscribed enhancers in TFBS	ER	LC	RC	*P*	P_{adj}
A549Pol2Pcr2xDex100nm	487	593	4.27	3.75	4.86	2.89E-102	1.11E-99
A549Pol2Pcr2xEtoh02	481	619	4.02	3.54	4.57	8.19E-95	1.58E-92
Mcf7Pol2Serumstim	363	357	5.14	4.41	6.00	1.95E-92	2.50E-90
Mcf7Pol2Serumstvd	340	312	5.49	4.67	6.45	1.24E-91	1.19E-89
Ecc1Pol2V0416102Dm002p1h	345	350	4.96	4.24	5.80	2.47E-85	1.91E-83
Hepg2Pol2Pcr2x	424	544	3.98	3.47	4.55	1.91E-83	1.23E-81
Hepg2Pol2Forskln	370	424	4.41	3.80	5.11	1.12E-81	6.18E-80
A549Pol2	309	295	5.23	4.42	6.18	2.22E-80	1.07E-78
Mcf7Pol2	242	193	6.16	5.06	7.51	2.78E-72	1.07E-70
K562Pol2V0416101	407	596	3.45	3.02	3.95	3.96E-68	1.39E-66
Hepg2Pol24h8V0416102	313	396	3.92	3.35	4.58	7.76E-62	2.00E-60
Helas3Pol2	393	605	3.27	2.85	3.74	2.65E-61	6.39E-60
Nb4Pol2	310	406	3.78	3.23	4.42	5.40E-59	1.16E-57
Helas3Pol2	312	412	3.75	3.21	4.38	9.07E-59	1.84E-57
K562Pol2	324	448	3.59	3.08	4.17	7.47E-58	1.44E-56
Mcf10aesPol2Tam	373	578	3.23	2.81	3.71	1.66E-57	3.05E-56
K562Pol2	348	513	3.38	2.92	3.90	1.99E-57	3.34E-56
Hct116Pol24h8V0416101	559	1133	2.54	2.27	2.83	4.02E-57	6.47E-56
K562Pol2Ifng6h	267	318	4.12	3.48	4.89	2.89E-56	4.46E-55
Helas3Pol2s2Iggrab	233	244	4.66	3.86	5.63	1.15E-55	1.58E-54
Hepg2Pol2Iggrab	314	441	3.52	3.02	4.10	5.74E-55	7.63E-54
K562Pol2Ifng30	275	351	3.85	3.26	4.54	8.90E-54	1.14E-52

Mcf10aesPol2Etoh01	421	784	2.69	2.37	3.06	3.18E-49	3.32E-48
Hepg2Pol2	197	210	4.54	3.70	5.56	2.48E-46	2.18E-45
Helas3Pol2Pcr1x	466	974	2.40	2.13	2.71	3.94E-44	3.31E-43
Hct116Pol2Ucd	213	262	3.94	3.26	4.76	2.18E-43	1.69E-42
K562Pol2Ifna30	214	280	3.70	3.07	4.46	1.22E-40	9.04E-40
K562Pol2Ifna6h	218	297	3.55	2.96	4.27	2.11E-39	1.43E-38
Panc1Pol24h8V0416101	202	344	2.82	2.35	3.38	3.37E-27	1.46E-26
K562Pol24h8V0416101	298	650	2.22	1.92	2.57	1.19E-25	4.92E-25
K562Pol2s2Iggrab	75	67	5.26	3.72	7.44	2.99E-21	1.00E-20
K562Pol2b	165	307	2.56	2.09	3.11	1.75E-19	5.36E-19
K562Pol2Iggmus	221	490	2.16	1.82	2.55	1.88E-18	5.42E-18
Hepg2P300V0416101	399	1114	1.74	1.54	1.97	5.61E-18	1.56E-17
A549Pol2s2Iggrab	49	37	6.18	3.95	9.77	3.88E-16	9.78E-16
T47dP300V0416102Dm002p1h	239	605	1.89	1.61	2.21	1.33E-14	3.14E-14
Hepg2Pol2s2Iggrab	53	52	4.76	3.18	7.13	2.35E-14	5.39E-14
Helas3Pol2b	78	120	3.05	2.25	4.09	7.63E-13	1.56E-12
K562P300Iggrab	409	1367	1.43	1.27	1.61	4.92E-09	8.32E-09
K562Pol2s2	17	14	5.62	2.61	12.33	3.67E-06	5.15E-06
A549P300V0422111Etoh02	372	1353	1.30	1.15	1.47	3.08E-05	4.14E-05
Helas3P300sc584sc584Iggrab	369	1961	0.85	0.75	0.96	7.09E-03	8.58E-03
K562P300	39	119	1.52	1.03	2.20	2.80E-02	3.26E-02

Abbreviations: TFBS: trascriptional factor binding sites; ER: enrich ratio; LC: left confidence; RC: right confidence
